# Supplementary material for: Patterns of Intron Gain and Loss in Fungi
Source: PLoS Biol. 2004 Nov 30;2(12):e422. doi: 10.1371/journal.pbio.0020422 (PMC532390; doi:10.1371/journal.pbio.0020422)
Supplement: Table S1 — Also available at http://genes.mit.edu/NielsenEtAl/. (4.3 MB ZIP). [file pbio.0020422.st001.zip › NielsenEtAl/html/843.html]

AN6443.1.NCU04161.1.MG08309.1.FG11028.1


```
 CLUSTAL W (1.82) Multiple Sequence Alignments - Introns Inserted


Sequence 1: NCU04161.1	1464 aa
Sequence 2: MG08309.1	1505 aa
Sequence 3: AN6443.1	1396 aa
Sequence 4: FG11028.1	1382 aa
Alignment Length: 1572 aa
Number Identitical Residues: 347 aa
Alignment Score (without introns) 23904


MG08309.1 	MAGPPPTHGPGSRSSASNDPDIRRQTQDDSEQVVSDATMSRDADLIRPSAPSPAPKPSAD
NCU04161.1	MAEFP----------------------DEKEKEIS-----------LPNPVADQRHRDDE
FG11028.1 	------------------------------------------------------------
AN6443.1  	------------------------------------------------------------
          	                                                            

MG08309.1 	LDDATSSDEEKHQRQSSSESLSLPATATRNGLNRTKSTATDVSATYSTHLRTLQQEWTSR
NCU04161.1	IEEAHRSDED-VEKIAIGSSVVEENDEKRAELNRMRTNATNTSVTT---AATIHPHVKPK
FG11028.1 	------------MATNDGKSFPPRKWYRRVPLLSTTPIKQRLTAPK--------------
AN6443.1  	------------MRLSSEAKIAESGGQPPTAGSRSETGSESTEAES--------ADPKAQ
          	                   ..               .      .             .. 

MG08309.1 	PWYRK-LNPLRWGGIPPLPDERAPCPEVDAGFLSKLLFSWQGPLMK~TGYRRSLQLTDIW
NCU04161.1	PWYKQ-PNPLRWGKIAPIPETRRPSPEYNAGFFRSLFFSWMGPLMT0------LELNDIY
FG11028.1 	----SSPSPANVEDQNNGHHDKLPLPEANANWLSVLTFNWLGRLLR~TGYTRPLQTDDLY
AN6443.1  	KWYQRSLNPLRWQKIPPVPEERTVSREYGASFFSIASFQWMAPLMK0VGYLRPLELQDIW
          	     : .* .        . :    * .*.::    *.* . *:   .   .*:  *::

MG08309.1 	AVNPDRAVEPMSDRVRASFKKRVAAG-----------------------QRFPLALALHE
NCU04161.1	QVNPARSVDPLTERMRESYKRRVEKG-----------------------DKYPLLWAMHE
FG11028.1 	DMPSHRLVEDHSNRLEEAWAARLATNRGRKPVNSKNPLSWRPFWARHTTDTTVLTLALND
AN6443.1  	TVNPDREVDVLTKRFEVSLEKRTNAG-----------------------AKRPLLWALYD
          	 : . * *:  :.*.. :   *   .                           *  *: :

MG08309.1 	AFFREFWLGGFCSLISTLMQVLSPFMLRFLIQFATDAYNAANEGAPAPPVGRGAGLIVGV
NCU04161.1	TFFWEFWIGGMCQLAASILQVMSPFTLRYLIQFATNAWVATHSGAPPPGIGSGLGLVFGI
FG11028.1 	VCFKWFWIGGILKLAGDLSQIISPLLIRFLIGTLSDSSQQ--------GLRSGFGYAVGL
AN6443.1  	TFRFEFLLGGFCHLISSLLIVFAPYLTRYQIAFATEAYVAQRSGQPAPRIGRGMGFVVGI
          	.    * :**:  * . :  :::*   *: *   :::     .. ... :  * *  .*:

MG08309.1 	VLMQIAQSLAMNHFIYHGMICGGQSRATLIQMVYEKSMVLSGRAKAGGSKDNNN----ET
NCU04161.1	TAMQILQSLCINHFIYRGMLIGGMARASLISLIYEKSMVISGRAKAGGADASDVPAAKAA
FG11028.1 	FLLLVFSVTSNVHGFYRSYTTGILLRGALMHVIYRRATTQLS------------------
AN6443.1  	TVMQAIQSLCTNQFLYRGQMVGGQIRAVLILQIFNKAMKLSGRAKAGGVQSPEQ-----Q
          	  :   .  .  : :*:.   *   *. *:  ::.::    . :.:.. .  .       

MG08309.1 	KDNKENKDKKKKGSKGGVNPEGQGAGWANGRI~VNLMSVDTYRVDQAFGLFHIIWTAPLA
NCU04161.1	AEKDAKKKSKKKGKKGQAGVEGDGAGWGNGRI0INLMSVDTYRVDQASGLFHIIWTAPVS
FG11028.1 	------------------ERAKLKHGLGTGKL~MSLISADVTRVDFCCGYFHVAWTSIFQ
AN6443.1  	EKIKELKAAKDQALKKPGSPPADDKGWGNGRI~VALMSIDVDRINLACGMFHISWTAPVS
          	 . .  .  .... .          * ..*:: : *:* *. *:: . * **: **: . 

MG08309.1 	CIITLIVLVINITYSALAGFALLVIGVPILTRAIKSLFIRRKGINRITDQRVSLTQEILQ
NCU04161.1	IIITLVLLLVNLTYSALAGFALLIIGIPVLTKAIKSLFARRKAINKITDQRVGLTQEILQ
FG11028.1 	MLLCFALTVWTMGYSALPGFGLLALLYPLQSFMMRRLLLLRRGSMPFTDARVKAVVEAVS
AN6443.1  	IIVALILLLVNLTYSALAGFGLLVIGMPFLTYAVRFLFKRRRNINKLTDQRVSLTQEILQ
          	 :: : : : .: ****.**.** :  *. :  :: *:  *:    :** **  . * :.

MG08309.1 	SVRFVKYFGWESSFIARLHELRAREISAIQMLLSIRNAILAVSLSLPIFASMLSFITYSL
NCU04161.1	SVRFVKFFGWESSFLKRLQEFRDREVSAIQVLLALRNAIMAISISLPIFASMLAFITYSL
FG11028.1 	AIRLVKTNAYEESLLSKIGKLRADEVVYIRKRMLLRALNTAISYTAPTLASVVSIVCYGA
AN6443.1  	GVRFVKFFGWESSFLDRLKEIRHHEIRSIQTLLAVRNGILCVSMAIPVFASMLSFITYAL
          	.:*:**  .:*.*:: :: ::*  *:  *:  : :*    .:* : * :**::::: *. 

MG08309.1 	SG-HGLNPAQIFSSLALFNGLRMPLNLLPLVIGQITDGWSSLKRVEEFLLAEEQNEDVVR
NCU04161.1	TN-HGLAPAKVFSSLALFNGLRMPLNMLPLVIGQVTDAWSSISRIQDFLLSEEREDEAII
FG11028.1 	TSKNGMDAGVVFSGLAYFMLLRTPLMALPTALSAVADARAALERLAMFMLASDVVADNQK
AN6443.1  	SN-HVLDPAPIFSSLALFNSLRMPLNLLPLVLGQITDAWTALNRIQEFIVAEEQKEDIER
          	:. : : .. :**.** *  ** **  ** .:. ::*. :::.*:  *:::.:   :   

MG08309.1 	RMDGENAIEMHGASFTWEKSPTQKKD------GEKEKKPVAAAGKEKPAPENGDGTADGP
NCU04161.1	KPDAPNAIEVHDASFTWERTPTQENESTVGGAGPKSKPEKGAKGKPKDVEAATPPSGDDS
FG11028.1 	SDFQSDLGYSSEMPE-------------------SNAVIRVEDATFAYHDDEQLLAADEK
AN6443.1  	DEHMPEAVRMDRASFTWER---------------KAADKEAEKVEKKANPRRTEPKSEAP
          	     :       . : .                .                     .:  

MG08309.1 	LTETE-REPFHLEELNLAIGRNELIAVIGTVGSGKSSLLAALAGDMRKTAG------EVV
NCU04161.1	STLVEEQEPFKLQDLNFTIGRNELVAVIGSVGSGKTSLLSALAGDMRKTSG------EVV
FG11028.1 	REPETSAATQRLWLDSFLVKRNQLVCIVGAVASGKSSVLKALLGDLQLVQARSFHLGLDK
AN6443.1  	TDSAESDEPFQLRDMTLDIRRDELVAVIGTVGSGKSSLLAALAGDMRLTDG------SVR
          	     .  . :*   .: : *::*:.::*:*.***:*:* ** **:: . .         

MG08309.1 	LGALRAFCPQYSWIQNTTVRDNILFGKEMDESWYREVIKA2NCYGRCALEPDLDMLPNGD
NCU04161.1	LGAQRAFCPQYAWIQNATLKDNILFGKEMDPEWYRDVIKA~-----CALQPDLDMLPNND
FG11028.1 	SSSQIAFAPQSAWLLSETVRENIVFGRPFDHAWYNEVLTR~-----CCLHPDMKVLPDGD
AN6443.1  	LSTSRAFCPQYTWIQNTSLRDNILFGKDYDEKWYDQVIDA~-----CALKPDLEILPNGD
          	 .:  **.** :*: . ::::**:**:  *  ** :*:        *.*.**:.:**:.*

MG08309.1 	LTEIGERGITISGGQKQRLNIARAIYFDADMVLMDDPLSAVDAHVGRHIFDNAILGLLGD
NCU04161.1	LTEIGERGITISGGQKQRLNIARAIYFDADIVLMDDPLSAVDAHVGRHIFDNAILGLLKD
FG11028.1 	MTVVGENGVSLSGGQKQRISLARAIYGRSSLLFLDDCFSALDAHVGARVFDMVVNQARRN
AN6443.1  	ATEIGERGITISGGQKQRLNIARAIYFNAELVLLDDPLSAVDAHVGRHIMDKAICGLLKG
          	 * :**.*:::*******:.:*****  :.::::** :**:***** :::* .:     .

MG08309.1 	K--CRILATHQLWVLNRCDRIVWMEAGKIQAVGTFDDLVHNHEGFKQLMETHALEEKKDG
NCU04161.1	K--ARILATHQLWVLNRCDRIIWMDGGRIQAVDTFDNLMRDSEEFRQLLESTAQEEKKD-
FG11028.1 	NEGTIVLVTHSIRFARQADHIVYMEKGRISEQGSYEQLQNLDGAFTQFAGSQPLIADSDS
AN6443.1  	R--CRILATHQLHVLSRCDRIVVMDDGRIHAVGTFDELSRDNDLFKQLMSTASQDSKEDE
          	.    :*.**.: .  :.*:*: *: *:*   .::::* .    * *:  : .   ..* 

MG08309.1 	KKADDESAGDGEDTKDAKEKQPG-------DIKLKKGKSLMQTEEQAVASVPWSVYDDYI
NCU04161.1	-EAEAPAATSEEEAPKKK----------------KKAKGLMQAEERAVASVPWSVYTSYV
FG11028.1 	GSETASSHEDEQAVDEKVTEKEGPSSKGNDKPSTTRGKEVMQTEERVVGSVSGRTYIRYA
AN6443.1  	EEATEVVEEEAEKQAQQEP--------------TKPAAALMQQEEKATDSVGWTVWKAYI
          	 .       . :   .                  . .  :** **:.. **   .:  * 

MG08309.1 	RSSGSILNAPLTIALLLVAQGANIATALWLSYWTSDRFS-LPTPVYIGIYAGLAVAQVVL
NCU04161.1	KASGSYLNAPIVLVLLVISQGSNIMTSLWLSWWTSDKFG-LSLGQYIGAYAGLGAMQALL
FG11028.1 	QFGNAFLTVPLLLLSIAIYQGTSIVSPLWLSWWQNNQYTSISEDAYMGGYAAFGAGQSLG
AN6443.1  	RASGSYFNALAILFLLAFANVVNVWTNLWLSYWTSNHYPSLSTGQYIGIYAGLGAGSALT
          	: ..: :..   :  : . :  .: : ****:* .::: ::.   *:* **.:.. . : 

MG08309.1 	LFGFMVALSVLGTRASRTMLHQAVTRVLRAPMSFFDTTPLGRITNRFSRDVDVMDNNLAD
NCU04161.1	MFAFMVSLSMFGTTASKNMLRQATFRVLRAPMSFFDTTPLGRITNRFSRDVDVMDNNLTD
FG11028.1 	LFCMSATFALFCFWCSNKLHHASMSKVLLAPVAFFDTTPQGRITHRFSKDVDAVDNVVGE
AN6443.1  	MFIFSTYMSTAGTNASRQMLQLAMTRVLRAPMSFFDTTPLGRITNRFSKDIGVMDNELCD
          	:* : . ::     .*. : : :  :** **::****** ****:***:*:..:** : :

MG08309.1 	AMRMYFFSVSGILSTFALIIAFFHYFAAALVPLFFVFLASTAYYRASAREVKRFESTLRS
NCU04161.1	ALRMYFFSIGAIISTFALIIAYFYYFAIALVPLFTLFLFATGYYRSSAREVKRFEAVLRS
FG11028.1 	TLRLFISTFVQVLGTIIVVSIIVPAFLAIAFALLLIYTWTGMYYRPSSRELRRLNNLLRS
AN6443.1  	AMRMYAITITMIVSIMILIIVFYHYFAIALVPLFLLFLTASNYYRSSAREMKRHESILRS
          	::*::  :.  ::. : ::      *    ..*: ::  :  ***.*:**::* :  ***

MG08309.1 	TLFAKFSEGLSGVACIRAYGLQDRFAADLRAAIDDMNSAYYLTFSNQRWLSIRLDAIGNA
NCU04161.1	TVFAKFNEGLSGVASIRAYGLQNRFVEDMRKAIDDMDSAYFLTYSNQRWLSTRLDMIGNA
FG11028.1 	RIYEHFGESLSGLPTLKAFGAVPRFVDDNAHKIDTENTAYWLSVACQRWLNLRLDLCGAT
AN6443.1  	AVYARFSEAITGTASIRAYGVQNQFRSSLRDSVDTMNGAYFLTFSNQRWLSVRLDAVAVL
          	 :: :*.*.::* . ::*:*   :*  .    :*  : **:*: : ****. ***  .  

MG08309.1 	LVLTTGVLVVTNRFDVPPSIGGLVLSYILSIVQMIQFTVRQLAEVENGMNAVERLRYYGR
NCU04161.1	LVFTTGILVVTSRFSVNPSIAGLVLSYILAIVQMIQFTVRQLAEVENGMNAVERLLYYGT
FG11028.1 	LVLAAGLLVVGLRGTIDPSSGGVVLSYIVTAQAVFGNMIRQSAEIENNMNSVERMLHYAY
AN6443.1  	LVFVTGVLVVTSRFDVSPSISGLVLSYILAIAQMLQFTVRQLAEVENNMNATERVHYYGT
          	**:.:*:***  *  : ** .*:*****::   ::   :** **:**.**:.**: :*. 

MG08309.1 	ELESEAPLKT-----IEVAPSWPQKGEIIFEDVEMRYRPGLPLVLRGLDMKVRGGERIGI
NCU04161.1	QLEEEAPSKT-----IDVRPSWPEKGEIIFDNVEMRYRAGLPLVLQGLNVHIQGGERIGI
FG11028.1 	NIEQEPEHRVERVDKELETSQWPSAGHVQFQSFTLSHRPGLDPALKDINLDIKPGEKVGV
AN6443.1  	QLEEEAPAHIP---SNPVPESWPPHGEITFDNVAMRYRPGLPLVLKNLSMNISGGERIGI
          	::*.*.  :     .     .**  *.: *:.. : :*.**  .*:.:.:.:  **::*:

MG08309.1 	VGRTGAGKSSI~MSALFRLVELSGGRITIDGLDIATVGLGDLRSRLAIIPQDPTLFKGTV
NCU04161.1	VGRTGAGKSSI~MSTLFRLVEISGGHITIDGIDISTIGLQDLRSRLAIIPQDPTLFRGTV
FG11028.1 	VGRTGAGKTTL1ISALLRITEPTNGRILIDGVDIHTVGLHLLRSNLSVISQDAVLLAGTI
AN6443.1  	VGRTGAGKSSI~MSALFRLTELSSGRITIDGVDISTIGLHDLRSRLAIIPQDPTLFRGSI
          	********::: :*:*:*:.* :.*:* ***:** *:**  ***.*::*.**..*: *::

MG08309.1 	RSNLDPFGEHTDLELWDALRQADLVG-------PETGSGTGTPTAFQEKEAGGGGGG--R
NCU04161.1	RSNLDPFGEHTDLELWSALRQADLVQDDQATTTTATPSASGNALVVAETPAASNGNSNNR
FG11028.1 	RYNLDPFQQYDDEWLRQCLDRVGLSQ--------------ISYHTDAEKQDSDSSGDAPS
AN6443.1  	RSNLDPFNEHSDLELWDALRKAHLID-----------SDTKDSAVDASNPNGNANAQ--R
          	* ***** :: *  * ..* :. *             : :    .  ..  .. .     

MG08309.1 	IQLDTVVEEDGLNFSLGQRQLMALARALVRGSQIIICDEATSSVDMETDAKIQATMAVGF
NCU04161.1	IGLDSVVEEDGLNFSLGQRQLMALARALVRGSQIIVCDEATSSVDMETDDKIQRTMASAF
FG11028.1 	LNLNSEVKENGSNISQGQRSLISIARALVRKSKIVILDEATASIDGRSDAKLQAMLNEVV
AN6443.1  	LTLDTAVDEEGLTFSLGQRQLMALARALVRNARIIICDEATSSVDFATDQRIQETMAQGF
          	: *:: *.*:* .:* ***.*:::****** ::*:: ****:*:*  :* ::*  :   .

MG08309.1 	RGKTLLCIAHRLRTIVG-YDRICVMDQGRIAELGEPAELFKMEGGIFRGMCERSGIGERD
NCU04161.1	RGKTLLCIAHRLRTIIN-YDRICVMDKGRIAEIGTPMELFEMEGGIFRGMCERSGIRAED
FG11028.1 	GDATVLTVAHRLDTIVSTCDKVVVMDSGRVVEFATVSELYAQPGSIFRSLCDAAKISL--
AN6443.1  	EGKTLLCIAHRLKTIIH-YDRICVMDQGSIAEIDTPLNLWEKEDGIFRAMCERSGISRED
          	 . *:* :**** **:   *:: ***.* :.*:    :*:   ..***.:*: : *   .

MG08309.1 	ILAAREGLGIAGGVDE
NCU04161.1	IRLAREIVDNDRAL--
FG11028.1 	----------------
AN6443.1  	IVGQVEKE--------
          	     .
```
